# Preclinical therapeutic efficacy of a novel blood-brain barrier-penetrant dual PI3K/mTOR inhibitor with preferential response in PI3K/PTEN mutant glioma

**DOI:** 10.18632/oncotarget.15566

**Published:** 2017-02-21

**Authors:** Dimpy Koul, Shuzhen Wang, Shaofang Wu, Norihiko Saito, Siyuan Zheng, Feng Gao, Isha Kaul, Masaki Setoguchi, Kiyoshi Nakayama, Kumiko Koyama, Yoshinobu Shiose, Erik P. Sulman, Yasuhide Hirota, W.K. Alfred Yung

**Affiliations:** 1Brain Tumor Center, Department of Neuro-Oncology, The University of Texas MD Anderson Cancer Center, Houston, Texas, USA; ^2^ Brain Tumor Center, Department of Bioinformatics and Computational Biology, The University of Texas MD Anderson Cancer Center, Houston, Texas, USA; ^3^ Medicinal Chemistry Research Laboratories, Daiichi Sankyo Co. Ltd., Tokyo, Japan; ^4^ Drug Metabolism & Pharmacokinetics Research Laboratories, Daiichi Sankyo Co. Ltd., Tokyo, Japan; ^5^ Oncology Research Laboratories, Daiichi Sankyo Co. Ltd., Tokyo, Japan; ^6^ Department of Radiation Oncology, The University of Texas MD Anderson Cancer Center, Houston, Texas, USA

**Keywords:** PI3K/mTOR/ brain penetrant, PI3K mutations and/or PTEN aberrations

## Abstract

Glioblastoma (GBM) is an ideal candidate disease for signal transduction targeted therapy because the majority of these tumors harbor genetic alterations that result in aberrant activation of growth factor signaling pathways. Loss of heterozygosity of chromosome 10, mutations in the tumor suppressor gene PTEN, and PI3K mutations are molecular hallmarks of GBM and indicate poor prognostic outcomes in many cancers. Consequently, inhibiting the PI3K pathway may provide therapeutic benefit in these cancers. PI3K inhibitors generally block proliferation rather than induce apoptosis. To restore the sensitivity of GBM to apoptosis induction, targeted agents have been combined with conventional therapy. However, the molecular heterogeneity and infiltrative nature of GBM make it resistant to traditional single agent therapy. Our objectives were to test a dual PI3K/mTOR inhibitor that may cross the blood–brain barrier (BBB) and provide the rationale for using this inhibitor in combination regimens to chemotherapy-induced synergism in GBM. Here we report the preclinical potential of a novel, orally bioavailable PI3K/mTOR dual inhibitor, DS7423 (hereafter DS), in *in-vitro* and *in-vivo* studies. DS was tested in mice, and DS plasma and brain concentrations were determined. DS crossed the BBB and led to potent suppression of PI3K pathway biomarkers in the brain. The physiologically relevant concentration of DS was tested in 9 glioma cell lines and 22 glioma-initiating cell (GIC) lines. DS inhibited the growth of glioma tumor cell lines and GICs at mean 50% inhibitory concentration values of less than 250 nmol/L. We found that PI3K mutations and PTEN alterations were associated with cellular response to DS treatment; with preferential inhibition of cell growth in PI3KCA-mutant and PTEN altered cell lines. DS showed efficacy and survival benefit in the U87 and GSC11 orthotopic models of GBM. Furthermore, administration of DS enhanced the antitumor efficacy of temozolomide against GBM in U87 glioma models, which shows that PI3K/mTOR inhibitors may enhance alkylating agent-mediated cytotoxicity, providing a novel regimen for the treatment of GBM. Our present findings establish that DS can specifically be used in patients who have PI3K pathway activation and/or loss of PTEN function. Further studies are warranted to determine the potential of DS for glioma treatment.

## INTRODUCTION

PI3K/Akt/mTOR is a frequently dysregulated pathway in cancer and is activated by various mechanisms in GBM [1–5]. mTOR and class I PI3K are two major and interdependent oncogenic kinases that contribute to cancer biology through the synthesis of cellular components and the regulation of growth, proliferation, migration, survival, and angiogenesis. Aberrant activation of these pathways has been linked to cancer development and is frequently detected in malignancies. In the past decade, researchers have made progress in understanding the molecular pathogenesis of GBM, the sequential accumulation of genetic aberrations, and the dysregulation of growth factor signaling pathways. In about 15% of GBM tumors, PI3K plays a central role in cancer growth, survival, motility, metabolism, and angiogenesis. Another 40% of GBM patients lack a functioning PTEN gene, which normally shuts off the PI3K pathway [5].

Preliminary evidence suggests that PI3K pathway activation and PTEN inactivation indicate poor prognostic outcomes in many cancers and that inhibiting the PI3K pathway may provide therapeutic benefit in those same cancers. The recent development of new pharmacological compounds directed targeting PI3K, mTOR, or both have been developed, and some of them are currently being tested in humans in phase I/II trials [6]. Treatment of recurrent malignant gliomas with mTOR inhibitor monotherapy is ineffective [7, 8]. Several of these mTOR inhibitors have been or are currently being tested in the clinical trials setting specifically in gliomas, including temsirolimus, everolimus (RAD001), and sirolimus. Temsirolimus (CCI-779) has been the most extensively studied drug in clinical trials. Phase II trials with CCI-779 as a monotherapy in recurrent GBM showed no effectiveness despite low toxicity and initial disease stabilization [8]. BKM-120 (Buparlisib) a dimorpholino pyrimidine derivative is an oral pan-class I PI3K inhibitor that penetrates the blood-brain barrier (BBB) [6]. It is in clinical trials for solid tumors including GBM, and has anti-proliferative and pro-apoptotic effects in GBM cell lines independent of PTEN or EGFR status. Because of the promise of combination therapy, currently there is a significant emphasis on dual PI3K/mTOR inhibitors. Several novel small-molecule inhibitors have been developed that have dual specificity for these targets. Preclinical evaluations of dual PI3K/mTOR inhibitors, such as PI-103 and NVP-BEZ235, have demonstrated efficacy in blocking the growth of GBM cells *in vitro* and *in vivo* [9]. Rapamycin analogues mostly target mTORC1 and trigger a feedback loop, possibly through mTORC2, that activates Akt [10, 11]. One of the factors contributing to the failure of rapalogues may be their inability to fully access their target [12]. XL765- a PI3k/mTOR dual inhibitor has recently been shown to reduce cell viability *in vitro* and in limited animal study showed a possible effectiveness when combined with TMZ therapy [13]. Similarly PKI-587 and PKI-402 were shown to have a strong *in vitro* antitumorigenic effect across multiple cell types including glioma cells, while also slowing tumor growth in xenograft models [5, 14]. Another dual PI3K/mTOR inhibitor, PI-103, which is known to have monotherapy efficacy in glioma [5] was recently shown to specifically reduce tumor volumes in combination with NSC-delivered s-trail in an orthotopic intracranial xenograft model [15]. GDC-0084 is a potent, oral, selective, brain-penetrant small molecule inhibitor of phosphoinositide 3-kinase (PI3K) and mammalian target of rapamycin (mTOR) kinase. PX-866 -a PI3K inhibitor was relatively well tolerated, however, this study also failed to identify a statistically significant association between clinical outcome and relevant biomarkers in patients with available tissue. AKT activation also contributes to resistance to chemotherapy in various cancer types, and therefore, inhibitors of the PI3K/Akt pathway have been used as single agents and in combination with chemotherapy to overcome chemotherapeutic resistance.

In this study, we studied a brain-penetrant dual PI3K/mTOR inhibitor, DS-7423, that can inhibit PI3K/mTOR signaling in a diverse panel of GBM and glioma initiating cell (GIC) lines at a brain-achievable concentration. DS causes PI3K pathway suppression in the brain and shows efficacy in intracranial models of GBM. Combinations of DS with temozolomide (TMZ) demonstrated a significant survival benefit in animal models of GBM, which provides a basis for clinical investigation of DS combined with TMZ.

## RESULTS

### Pharmacokinetics of DS7423

DS7423 inhibits PI3K/mTOR activity likely binding to the ATP binding cleft of these enzymes and was tested against class I PI3K and other kinases using an HTRF assay format and direct measurement of substrate phosphorylation, respectively. DS was most potent against p110α, but also inhibited the other isoforms of class I PI3K with the following order of potency (IC_50_): p110α (17 nM) > p110γ (249 nM), p110δ (262 nM) > p110β (1143 nM). Further characterization of DS showed that it poorly inhibited a representative panel of 227 kinases in biochemical assays since greater than 50% inhibition was seen only against 2 other kinases: mixed lineage kinase 1 (MLK1) and never-in-mitosis gene a (NIMA)-related kinase 2 (NEK2).

### Pharmacokinetics of DS in mice

**Figure 1:**
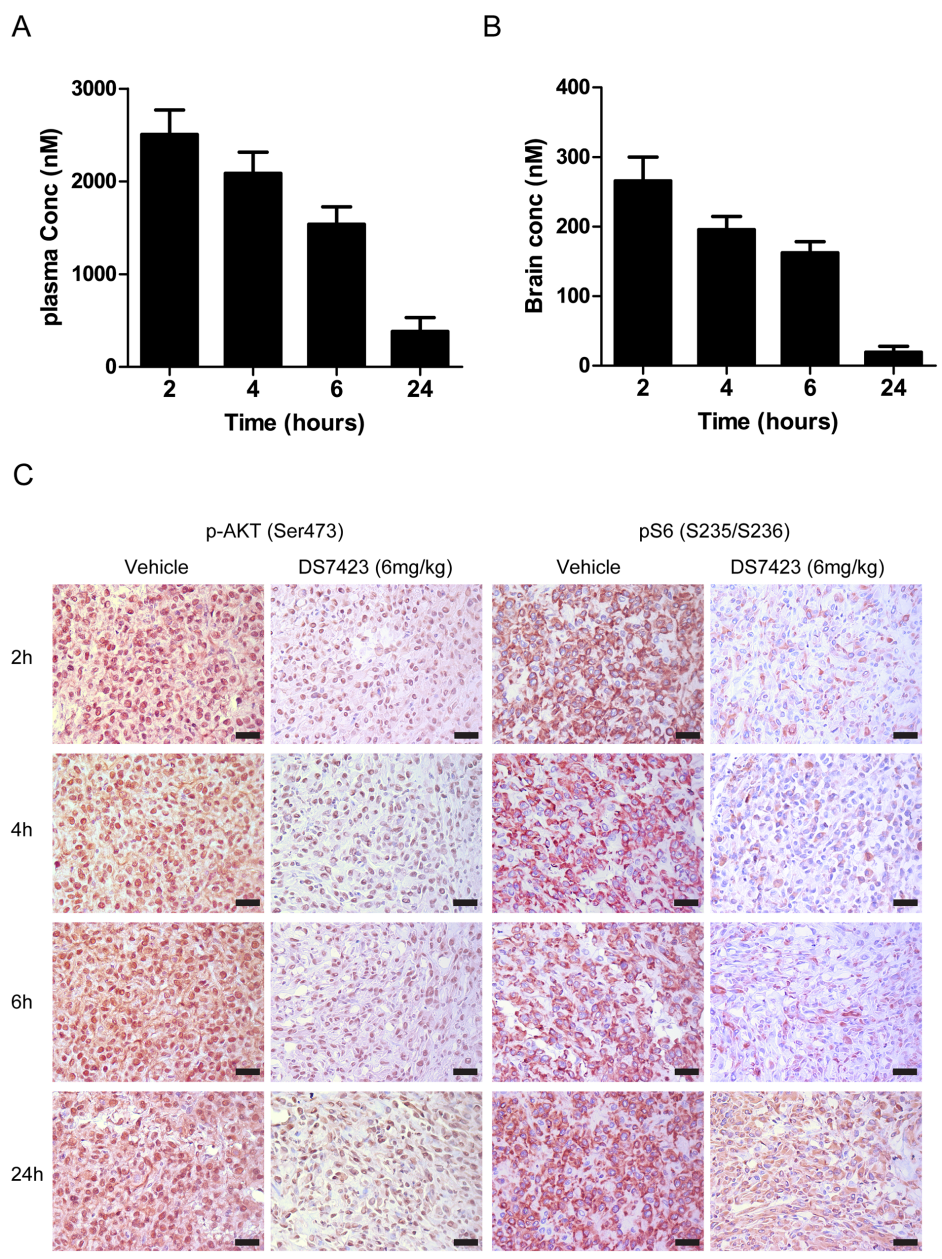
**A**. and **B**. Plasma and brain concentration-time profile of DS7423 following a single PO administration (6 mg/kg) to nude mice. **C**. Western blot of mouse brains probed with antibodies against pAkt (Ser473) and pS6 (Ser235/236) in a time profile similar to that shown in A and B. Results are presented as the mean ± SE of six animals in each group.

We studied the plasma concentration-time profile of DS following a single PO administration (6 mg/kg) in mice (Figure 1). Plasma concentrations remained generally constant and higher than 2 μmol/L up to 6 hours after treatment. The brain-to-plasma ratio of total concentrations remained unchanged and was approximately 0.1 between 1 and 6 hours after treatment (Figure 1A and 1B). Both plasma and brain DS concentrations decreased at 24 hours after treatment.

To correlate DS brain concentration with the modulation of PI3K/mTOR signaling in U87 tumors, we used immunohistochemical analysis to study the signaling alterations in tumor sections derived from mice at similar time points following a single PO dose of DS (6 mg/kg). Tissue sections were probed with antibodies against pAkt and pS6. DS caused a marked reduction in pAkt and pS6 staining in U87 tumors (Figure 1C) when compared with sections taken from untreated animals. DS plasma and brain concentrations decreased after 24 hours, which correlated with an increase in pAkt and pS6. These results indicate that DS affects the PI3K/mTOR pathway.

### DS disrupts PI3K signaling components in multiple glioma cells

Because a 200-nM DS concentration was achievable in the mouse brains, we sought to determine whether we could detect signaling alterations in a large cohort of GIC lines treated with this brain-achievable concentration. We analyzed the alterations of various PI3K signaling components by hierarchical clustering analysis of the RPPA data from GIC lines treated with 200 nM of DS by comparing patterns of protein expression to identify altered signaling events associated with DS response. DS treatment caused a decrease in signal intensity in PI3K/mTOR pathway proteins, specifically pAkt, pS6, pP70S6K, and p4EBP1 and PNDRG1 (Supplementary Figure 1).

### DS displays dose- and time-dependent effects on PI3K/mTOR signaling pathway

**Figure 2:**
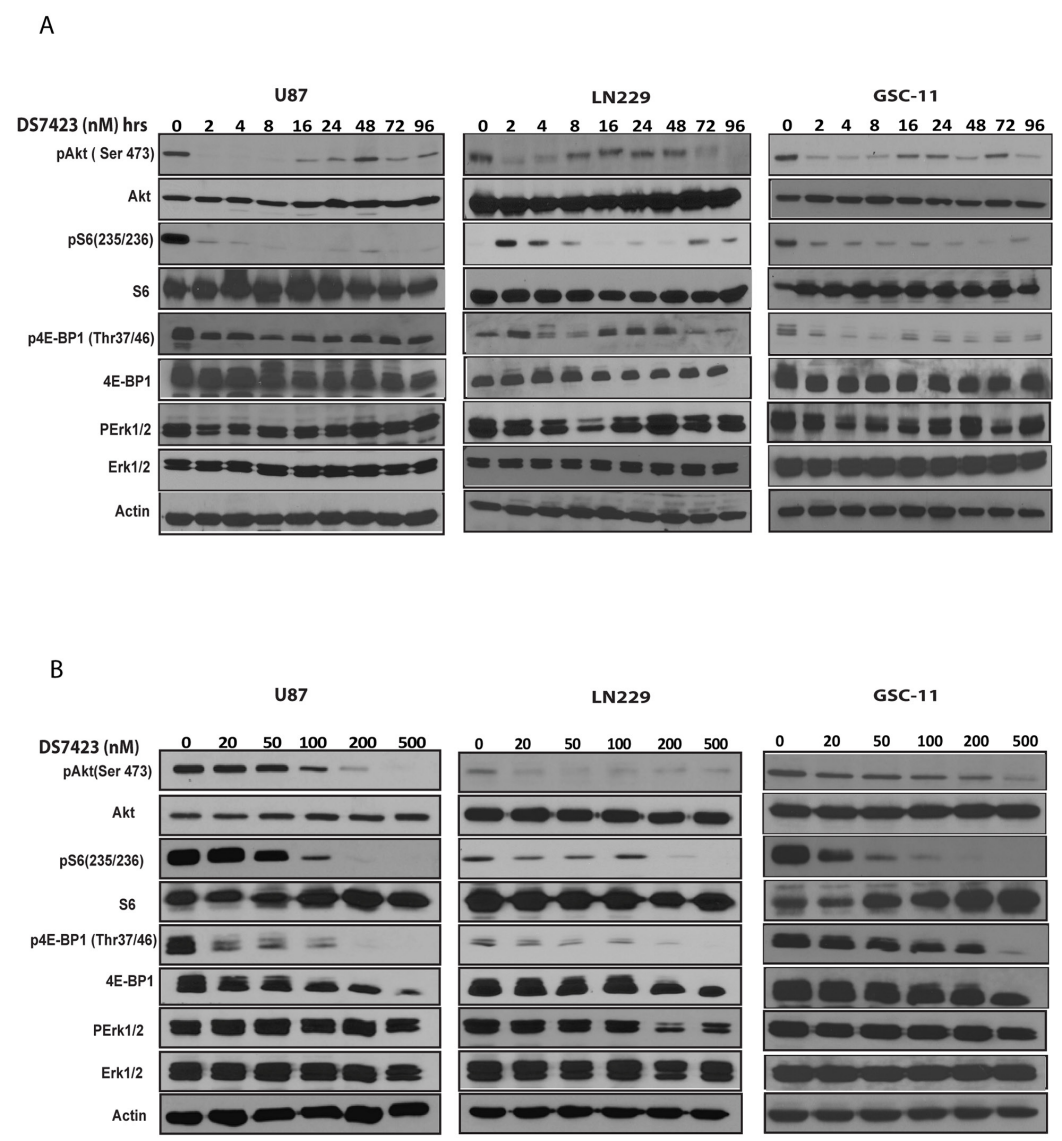
DS7423 suppresses the PI3K signaling pathway in a dose- or time-dependent manner **A**. and **B**. Western blotting was performed to analyze the cellular protein levels of PI3K signaling proteins in U87 and LN229 glioma cells and GSC11 GICs. Cells were treated with the indicated doses of DS7423 for the indicated time intervals. DS7423 inhibited phosphorylation of the PI3K signaling components pAKT, pS6, and p4EBP1 in a time-dependent manner. D. Cells treated with the indicated doses of DS7423 for 24 hours. DS7423 inhibited phosphorylation of the PI3K signaling components pAKT, pS6, and p4EBP1 in a dose-dependent manner.

The levels of PI3K/mTOR signaling proteins, including pAkt, pS6 and P4EBP1 decreased in a dose- and time-dependent manner in all tested glioma cells (Figure 2A and 2B) as well as in the glioma tumor-initiating cell line GSC11 (Figure 2A and 2B). DS suppression of pAkt caused subsequent effects on Akt effectors such as 4EBP1 and S6 at 200-nM concentrations, and the maximum effects on Akt phosphorylation were observed 8 hours after 200 nM DS treatment.

### DS shows preferential growth inhibition in PI3K-mutant and PTEN-depleted cells *in vitro*

**Figure 3:**
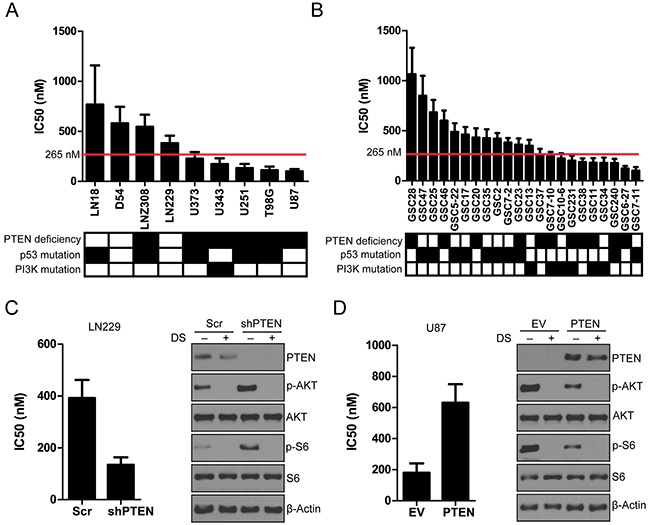
DS effectively inhibited the growth of glioma cells and GICs **A**. Waterfall diagram of IC_50_ of glioma cells and GICs. A panel of GIC lines was treated with various concentrations of DS. Cells were treated with increasing concentrations of DS in triplicate wells for 72 hours, and cell viability was assessed by a CellTiter-Blue assay. The results shown are from a single experiment with three independent replicates. The graph depicts cell viability at 72 hours. Cell viability in the vehicle control was considered to be 100%.

To determine the cytotoxicity of DS in GBM cells and GICs *in vitro*, we used a colorimetric ATP-based assay. All GBM cells and GICs demonstrated a concentration-dependent decrease in cell viability after 72 hours of exposure to DS. Values of the half maximal inhibitory concentration for GBM cells and GICs were assessed, and cells treated with a DS concentration within the range of 150-265 nM (Figure 3A and 3B) were considered sensitive to DS treatment. In an effort to understand the molecular predictors of response to DS, we determined whether key alterations in the PI3K pathway, such as PIK3CA/PIK3R1 mutations, or PTEN alterations were associated with greater sensitivity to DS.We found that cell lines harboring oncogenic mutations in PIK3CA/PIK3R1 and PTEN alterations were significantly more sensitive than cell lines without these alterations. To further determine the role of PI3K/PTEN in regulating cell proliferation in GBM lines, we knocked down PTEN in PTEN wild-type LN229 glioma cells and showed that depletion of PTEN in LN229 cells caused further growth inhibition when combined with DS treatment (Figure 3C). To investigate the role of PI3K/PTEN, we expressed PTEN in PTEN-mutant U87 glioma cells. We showed that PTEN introduction rescued the cell growth inhibition caused by DS treatment (Figure 3D). These data suggest that PI3K/PTEN alterations might be major contributors to sensitivity to DS treatment.

### Efficacy of DS in brain tumor models

**Figure 4:**
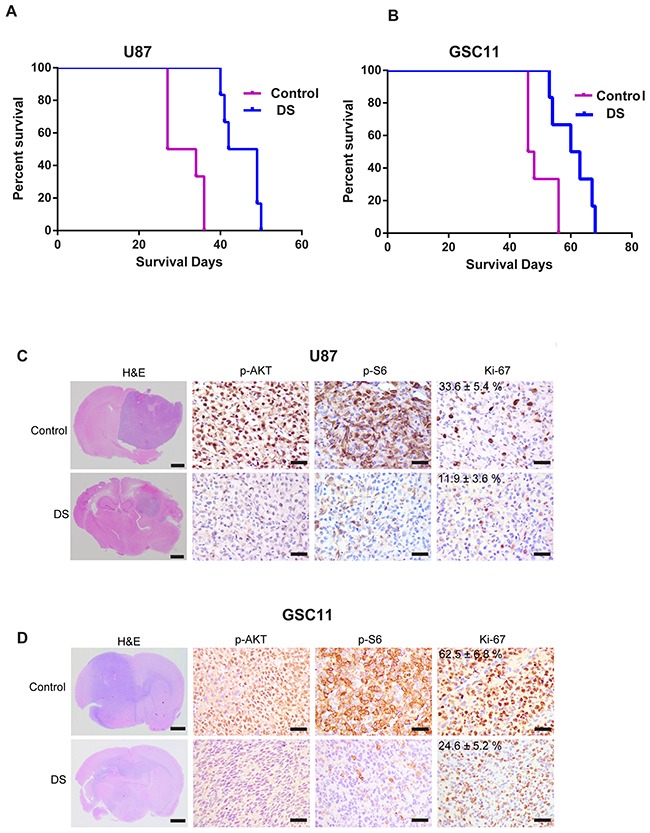
DS7423 suppressed tumor growth and prolonged survival in an orthotopic mouse xenograft model U87 glioma cells U87 and GSC11 GICs were used to generate orthotopic xenografts in mouse brains. Tumor-bearing mice received either vehicle or DS7423 at the indicated dose/schedules. Two mice were killed at the indicated time points (2 weeks and 4 weeks) after DS treatment. **A**. and **B**. Kaplan–Meier survival plots of tumor-bearing mice in vehicle or DS7423 treatment groups (n = 10). The log-rank method was used to test for a difference between groups. **C**. and **D**. Representative H&E-stained whole brain sections at 4 weeks after treatment; arrows indicate central necrosis. Immunostaining of the brain sections of mice treated with DS7423 for 4 weeks (n = 2). The tissue sections were incubated with antibodies against pAKT, pS6, and Ki-67. Bar, 100 μm.

We studied the efficacy of DS in 2 intracranial tumor models; the U87 glioma cell line and the GSC11 glioma initiating cell line are both PTEN mutant and GSC-11 is PIK3CA mutant cells. The median survival time of the U87 tumor-bearing control mice (i.e., those injected with methylcellulose only) was 30 days, whereas the median survival time of the U87 tumor-bearing mice orally treated with 6 mg/kg of DS was 46 days (Figure 4A; *P*=0.025). The median survival of the GSC11 tumor-bearing mice orally treated with 6 mg/kg of DS was 61 days (Figure 4B; *P*=0.014) compared with 47 days for the control-treated mice. The *in vivo* therapeutic efficacy of DS was assessed by plotting the Kaplan-Meier survival curves of the animals, and group data were compared using the log-rank test (Figure 4A and 4B). Histopathological staining revealed that DS treatment considerably reduced the tumor growth in U87 and GSC11 xenografts, as shown by decreased tumor mass in mice treated for 4 weeks with DS compared with those treated with vehicle control (Figure 4C). *Ex vivo* analyses of tumor tissues obtained from necropsied mice at the end of treatment showed marked inhibition of phosphorylated Akt and phosphorylated S6 in the DS-treated animals (Figure 4D). We also studied LN229 a PTEN wild type cell line to study the efficacy of DS in intracranial model. Results demonstrated that DS was ineffective in a LN229 tumor bearing mice and had no effect on median survival of animals clearly demonstrating that DS was ineffective in PTEN/PI3K wild type background (Supplemental Figure 2).

### DS synergizes with TMZ *in vitro* and enhances TMZ-induced autophagy *in vitro*

**Figure 5:**
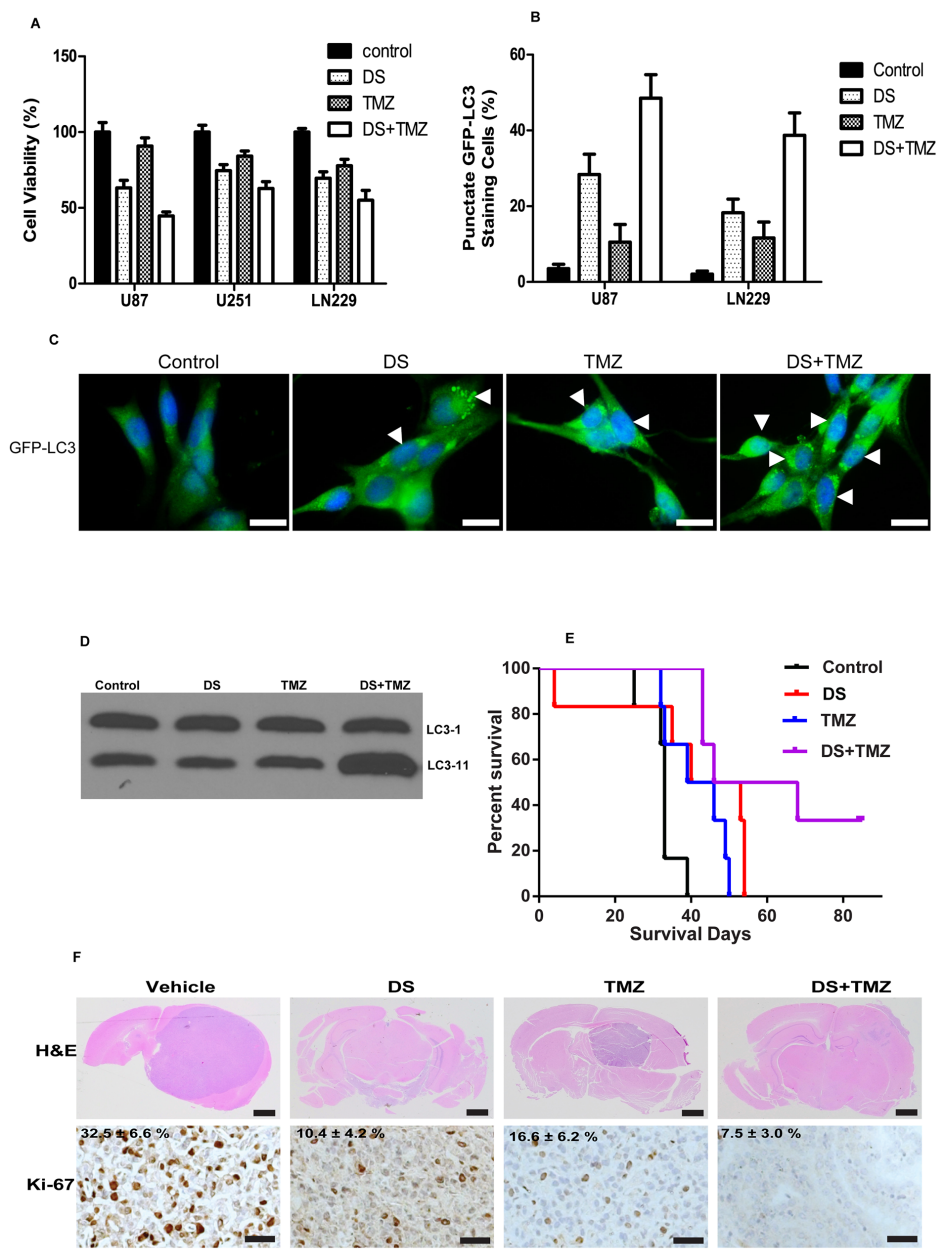
Combination activity of DS7423 and TMZ in GBM cells *in vitro* and *in vivo* **A**. Combination of DS7423 and TMZ resulted in additive cytotoxicity in 3 GBM cell lines in vitro. **B**. and **C**. The combination treatment of TMZ with DS increased autophagic cell death within 24 hours in U87 cells, as shown by increased punctate GFP fluorescence at 24 hours. Data are presented as the mean ± SD of three experiments. **D**. Cells treated with the either DS7423, TMZ or combination of DS and TMZ. DS and TMZ combination increased LC3-11 levels in comparison to either DS or TMZ alone. **E**. and **F**. Survival increased after oral administration of DS7423, TMZ, or DS7423 + TMZ in nude mice bearing intracranial U87 xenografts. D, Kaplan–Meier survival curves comparing the treatments using log-rank tests. DS and TMZ were compared with the control group, and DS + TMZ was compared with the control group, TMZ alone, and DS alone. E, Representative H&E-stained whole brain sections at 4 weeks after treatment. Immunostaining of the brain sections of mice treated with DS7423, TMZ, and DS+TMZ for 4 weeks (n = 2). The tissue sections were incubated with antibodies against Ki-67. Bar, 100 μm.

To investigate the ability of DS to modify the response of glioma cells to TMZ treatment, we evaluated the effects of TMZ (200 μM) alone, DS (200 nM) alone, and a combination of both TMZ and DS on the viability of the glioma cell lines U87 and U251 (Figure 5A). The cell viability after DS or TMZ treatment alone was 63% and 93%, respectively, but the combination of DS and TMZ reduced the cell viability to 44%, which clearly indicates that this combination of PI3K/mTOR inhibitors significantly increases cell growth inhibition.

To investigate the effects of DS, TMZ, or the TMZ/DS combination on autophagy in glioma cells, we transfected cells with GFP-LC3 constructs in addition to various drug treatment and control treatment regimens and examined the accumulation of punctate GFP-LC3 structures using fluorescence microscopy. During autophagy, LC3-II relocalizes to the autophagosomal membranes. Thus, the accumulation of GFP-LC3 puncta provides an effective way to detect autophagosomes. In control cells, GFP-LC3 punctate structures were not detected, but large numbers of GFP-LC3 punctate structures were found in cells treated with DS or TMZ (Figure 5C). Quantification of GFP-LC3 showed that TMZ (125 μM) or DS (100 nM) alone-induced autophagy in 10% or 30% of U87 cells, respectively, whereas the control cells had an autophagy rate of only 2%. Strikingly, the combination of DS and TMZ further induced autophagy in up to 50% of U87 cells. Western blot analysis of cell extracts treated with combination of DS and TMZ showed an increased LC3-11 protein in comparison to either control of single treatment of either DS or TMZ alone (Figure 5D). These results suggest that combination of DS and TMZ may cause autophagy in glioma cells.

### Combination of TMZ and DS increases mouse survival of U87 cells *in vivo*

To evaluate the long-term antitumor effect of the TMZ/DS combination, we further assessed the effects of TMZ, DS, or the combination treatment on the survival rate of U87 xenograft-bearing nude mice, which indirectly reflected the survival of tumor-bearing mice relative to their tumor burden. Our results showed that treatment with TMZ (7.5 mg/kg) alone did not reduce the tumor burden of the experimental mice compared with that of the control groups. However, the combination treatment with TMZ and DS significantly increased the survival rate of the mice compared with either agent alone (Figure 5E). Taken together, these results demonstrate that the combination of TMZ and DS can inhibit tumor growth and prolong the survival of mice bearing a U87 xenograft tumor, suggesting that the combination of TMZ with DS has a significant therapeutic benefit *in vivo*.

Immunohistochemical analysis of tumor tissues collected from the U87 xenograft-bearing nude mice treated with methylcellulose, TMZ, DS, or the TMZ/DS combination show moderate decrease in number of Ki-67-positive cells in TMZ or DS alone (Figure 5F). However, the TMZ/DS combination decreased the number of Ki-67-positive cells over that of either agent alone.

## DISCUSSION

Amplification or activating mutations of *PIK3CA*, the gene encoding the p110α subunit of PI3K, or *PIK3R1*, the gene encoding the p85 regulatory subunit of PI3K, have been found in approximately 15% of patients with GBM [18, 19]. Similarly, loss-of-function mutations, chromosomal deletions, or epigenetic gene silencing of *PTEN* have been found in approximately 40% of GBM cases [3] and have been shown to lead to poor survival [20]. There has been a tremendous effort to develop PI3K pathway inhibitors for the treatment of cancer. These include perifosine, Cal101, PX-866, and PI-103, with some PI3K inhibitors even being specifically assessed in GBM (XL765, XL147, and BKM 120). The results of many of these trials have been poor. One issue is the heterogeneity of GBM, with its redundant signaling inputs [21] and ability to bypass blockade of individual molecules through compensatory feedback loops [21]. Studies with very large sample sizes reporting that inhibition of mTOR can trigger a negative feedback loop that results in PI3K/Akt activation. Therefore, therapies using these PI3K/Akt/mTOR pathway inhibitors in combination with other inhibitors have recently become a focus. A major hurdle to inhibiting the PI3K pathway in the brain is the BBB, which excludes many therapeutic compounds and thus makes GBM treatment more difficult [22, 23]. DS, a PI3K/mTOR small molecule inhibitor is an, orally bioavailable currently in phase I clinical trials in patients with advanced solid tumors [24]. In our study, we examined the effects of DS in a panel of genetically diverse glioma cells and GIC, DS showed inhibition of PI3K/mTOR signaling in 9 glioma lines and 22 GIC lines. DS inhibited growth of glioma tumor cell lines and GICs at IC_50_ values of less than 250 nmol/L. To our knowledge, this is the first study to evaluate a PI3K/mTOR inhibitor in GBM using multiple distinct and previously characterized glioma cell lines and glioma-initiating stem cells is an important consideration when attempting to identify susceptible genotypes because of the genetic heterogeneity of GBM. We found that glioma models harboring alterations in PIK3CA/PIK3R1 and PTEN were highly sensitive to the antitumor effects of DS. Several studies have provided evidence to suggest that cancer cells harboring *PIK3CA* gain-of-function mutations are selectively sensitive to inhibitors of various components of the PI3K pathway. In a multicovariable analysis, treatment with a PI3K/AKT/mTOR inhibitor was the only independent factor predicting response to therapy in individuals harboring *PIK3CA* or *PTEN* aberrations [25]. In that study of 1,656 patients with advanced, refractory cancers tested for *PIK3CA* or PTEN abnormalities, *PIK3CA* mutations were found in 9% of patients, and PTEN loss and/or mutation was found in 13% of patients [25]. Therefore, screening for these mutations and aberrations can make treatments with PI3K/AKT/mTOR inhibitors more effective for patients.

Studies using various small molecule inhibitors targeting components of the PI3K signaling pathway are being evaluated in GBM patients [26], and the only brain areas affected were the areas where the BBB or blood–tumor barrier was permeable [27–31]. In this study, our group used the U87 glioma model to show that an impaired BBB results in almost 1/10^th^ penetration of DS in the brain in comparison to the plasma concentration following a single dose administration. The BBB-penetrant DS concentration was sufficient to prolong the median survival of mice with intracranial xenograft tumors without causing any obvious toxic effects. It is important to note that this penetrant DS was able to inhibit PI3K-mediated signaling in tumor tissue. We also studied a GSC11 GIC model of GBM and showed that DS could reduce tumor volumes and increase mouse survival, a finding supporting the use of DS as a safe and effective therapy for glioma. A similar dual PI3K/mTOR inhibitor (GNE-317) has also been identified and optimized to cross the BBB [32], that study mainly characterized the brain penetration, pathway modulation in the brain, and efficacy of GNE-317 in orthotopic xenograft models of GBM.

There is currently a significant emphasis on dual PI3K/mTOR inhibitors as targets for combination therapy. XL765 has recently been shown to reduce cell viability *in vitro*, and limited animal studies have shown possible effectiveness when combined with TMZ therapy [13]. Similarly PKI-587 and PKI-402 were shown to have a strong *in vitro* antitumorigenic effect across multiple cell types, including glioma cells, while also slowing tumor growth in xenograft models [33]. Another dual PI3K/mTOR inhibitor, PI-103, an effective monotherapy for glioma [34], was recently shown to specifically reduce tumor volumes in combination with Neural stem cell delivered s-trail in an orthotopic intracranial xenograft model [34]. PI-103 combination therapy has also proven effective in sensitizing cells to chemotherapy [35].

Bender and colleagues [35] showed that PI3K inhibitors synergize with various chemotherapeutics (doxorubicin, etoposide, topotecan, cisplatin, vincristine, and paclitaxel) to trigger apoptosis in neuroblastoma cells. In the current study, we combined DS with TMZ, an important component of multimodality GBM treatment. DS caused statistically significant TMZ sensitization both *in vitro* and *in vivo*, which warrants further evaluation of DS as part of a combination therapy regimen. Enhanced antitumor activity was observed in mice bearing established U87 xenografts treated with DS in combination with TMZ. This effect was accompanied by significantly increased survival in the combined therapy group compared with that of the groups treated with a single agent. The extended maintenance of tumor growth inhibition after chemotherapy by continuous treatment with DS supports further clinical investigation of these combinations.

In summary, our experiments demonstrate that DS is a brain-penetrant dual PI3K/mTOR inhibitor that exhibits potent anticancer activity as a single agent in preclinical glioma models and further enhances the effects of TMZ in human glioma xenografts, which supports the benefit of combining two therapeutic modalities that have entirely different mechanisms of activity. The enhanced activity of DS in combination with “standard of care” chemotherapeutic agents for glioma presents a compelling rationale for comparable clinical studies in this disease. DS is currently in phase 1 clinical trials in patients with advanced solid tumors.

## MATERIALS AND METHODS

### Cell lines and culture conditions

Nine well characterized GBM cell lines and 22 patient-derived GIC lines (provided by Drs. Frederick. F. Lang and Erik. P. Sulman, Brain Tumor center, M. D. Anderson Cancer Center) with various P53, PIK3CA, and PTEN statuses were used for this study. GBM cells were maintained as monolayer cultures and GICs were cultured as GBM neurospheres in DMEM/F12 medium containing B27 supplement (Invitrogen, Grand Island, NY) and basic fibroblast growth factor, and epidermal growth factor (20 ng/ml each).

### Reagents

**Figure 6:**
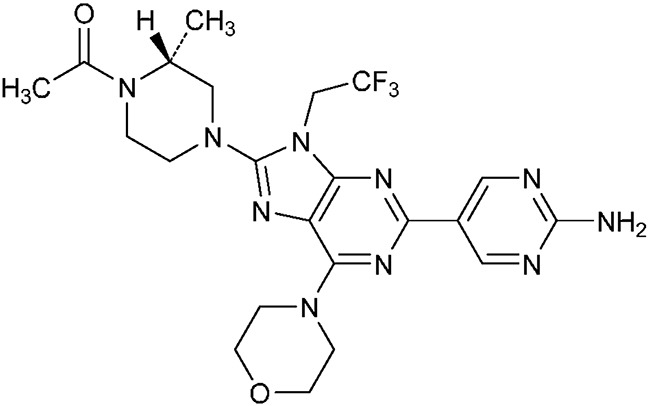
Chemical structure of DS-7423

DS74231-{(2R)-4-[2-(2-Aminopyrimidin-5-yl)-6-(morpholin-4-yl)-9-(2,2,2-trifluoroethyl)-9H-purin-8-yl]-2-methylpiperazin-1-yl}ethan-1-one (Figure 6) was provided by Daiichi Sankyo Co, Ltd. (Tokyo, Japan) For *in vitro* use, DS was dissolved in DMSO (Sigma-Aldrich Corp., St. Louis, MO) to a concentration of 10 mmol/L, stored at –20°C, and further diluted to an appropriate final concentration in DMEM at the time of use.

### Cell proliferation assay

The antiproliferative effect of DS on cells growing in culture was determined using the CellTiter-Blue (Promega, Madison, WI) viability assay. The mean drug concentration required to inhibit cell proliferation by 50% (IC_50_) compared with vehicle-treated controls was calculated using CalcuSyn Version 2.0 software (Great ShelfordCambridge, United Kingdom).

### Western blot analysis

Cells were incubated with DS and then harvested and extracts were prepared for immunoblotting analysis. The membrane was probed with the following primary antibodies: phospho-Akt (Ser473), phospho-Akt (Thr-308), total Akt, phospho-MAPK (Thr202/Tyr204), total MAPK, phospho-S6K1, total S6K1, phospho-S6, and total S6, anti-LC-3 (Cell Signaling Technology, Boston, MA). Anti-Δ-actin antibody was purchased from Sigma-Aldrich (St. Louis, MO).

### GFP-LC3 detection

U87 GFP-LC3 cells [16] were analyzed by using a fluorescence microscope, and the percentage of cells displaying GFP-LC3-positive puncta was quantified by counting 5 random fields in 3 independent experiments.

### Efficacy studies and biomarker analysis in an intracranial animal model

6 to 8-week-old male nude mice were obtained from the MD Anderson breeding facility and used in accordance with the Animal Care and Use Guidelines of MD Anderson Cancer Center. In this study, 5 × 10^5^ U87 (PTEN mutant) cells in DMEM/F12 serum-free media (5 μL) and GSC11 (PIK3CA mutant and PTEN mutant) were implanted intracranially into each mouse using a guide-screw system, as described previously [17]. Four days after injection of the tumor cells, mice were randomized into 2 groups (10 animals per group). Mice in-group 1 was given 6 mg/kg DS7423 via oral gavage, and mice in-group 2 were given the vehicle (0.5% methyl cellulose) used for administration of DS (control). Treatment frequency was once a day for 5 days with 2 days off between treatments, for a total duration of 4 weeks. Mice were monitored daily and euthanized when they became moribund. Whole brain was extracted for rapid freezing in liquid nitrogen and storage at –70° C.

Sections (5 μm thick) of formalin-fixed,paraffin-embedded whole brains from control vehicle- and DS-treated animal specimens were stained with anti-pAkt (Cell Signaling Technology), and anti-Ki67 antibodies (BD Biosciences, Franklin Lakes, NJ). The sections were visualized using a diaminobenzidinesubstrate kit, and the slides were examined under a bright-fieldmicroscope.

### Pharmacokinetic study in mice

Mice were implanted with U87 cells intracranially as described previously [17]. Twenty-one days after injection of the tumor cells,mice were randomized into groups. Mice were given a 6 mg/kg oral (PO) dose of DS. Blood samples of approximately 0.15 mL were collected from mice (*n* = 4 mice per time point) by retro-orbital bleeding in tubes containing K_2_EDTA as the anticoagulant pre-dose and at 2, 4, 6, and 24 hours post-dose. Plasma was collected and stored at −80°C until analysis. DS concentrations were determined by liquid chromatography-tandem mass spectrometry (LC-MS/MS) following plasma protein precipitation with acetonitrile and injection of the supernatant onto a TSK-GEL ODS-100V column (150 mm × 2 mm, 5 μm particle size). A Shimadzu 10A system (Shimadzu Corp., Kyoto, Japan) coupled with an AB Sciex API4000 triple-quadrupole mass spectrometer (Applied Biosystems, Foster City, CA) was used for the LC-MS/MS assay. The mobile phase was a mixture of acetonitrile, water, and 1M ammonium acetate (590:410:5, v/v/v) at a flow rate of 0.2 mL/min. Ionization was conducted in the positive-ion mode at a source temperature of 650°C using nitrogen nebulizing and heating gas. DS and its internal standard ([^2^H_8_]DS) were analyzed in the multiple reaction monitoring mode using the mass transitions of m/z 521→479 and 529→487, respectively. The lower and upper limits of quantitation were 2 and 5000-ng/mL10 μmol/L, respectively. Brains were collected at 2, 4, 6 and 24 hours post-dose from 4 animals at each time and stored at −80°C until analysis. For DS quantitation, mouse brains were homogenized in 3 volumes of 50% acetonitrile. The homogenates were further extracted with acetonitrile. LC-MS/MS analysis was conducted as described for the plasma. Brain homogenate concentrations were converted to brain concentrations for the calculations of brain-to-plasma ratios.

### Reverse-phase protein arrays (RPPA)

After DS treatment for 24 hours, cells were collected and lysed in lysis buffer. All samples were diluted to a final concentration of 1 mg/mL, and then 30 μL of each sample, arrayed in a series of dilutions, was printed in duplicate on slides. The slides were then subjected to immunostaining with a panel of 207 commercially available antibodies^13^. Slides were stained on an automated slide stainer (DAKO, Carpinteria, CA) using biotin-linked peroxidase-catalyzed signal amplification.

## SUPPLEMENTARY MATERIALS FIGURES AND TABLES



## Abbreviations

GBM: Glioblastoma
BBB: blood -brain barrier
GIC: glioma-initiating cell
PI3K: phosphoinositide 3-kinase
TMZ: temozolomide
