# Supplementary material for: Preclinical therapeutic efficacy of a novel blood-brain barrier-penetrant dual PI3K/mTOR inhibitor with preferential response in PI3K/PTEN mutant glioma
Source: Oncotarget. 2017 Feb 21;8(13):21741–53. doi: 10.18632/oncotarget.15566 (PMC5400620; doi:10.18632/oncotarget.15566)
Supplement: Supplementary file 1 [file oncotarget-08-21741-s001.pdf]

Preclinical therapeutic efficacy of a novel blood-brain barrier-penetrant dual PI3K/mTOR inhibitor with preferential response in PI3K/PTEN mutant glioma

SUPPLEMENTARY FIGURES

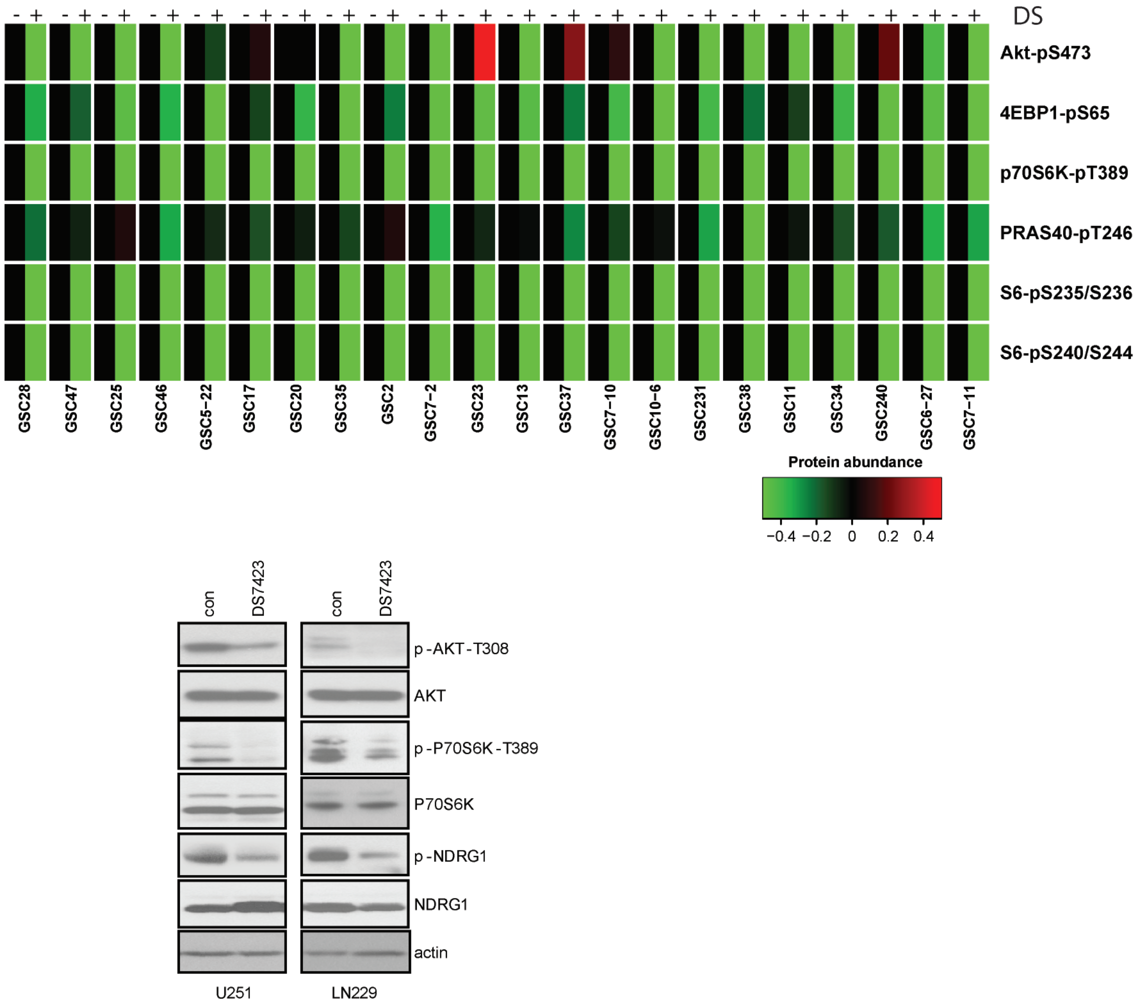

Supplemntary Figure 1: RPPA protein array analysis of glioma cells and GICs treated with DS7423. Color bars indicate an increase (red) or decrease (green) of signaling proteins following DS7423 treatment (200 nm0l/L, 24 hours). Differential changes in signaling proteins between untreated and treated GICs were selected with $P < 0.05$. Westorn Blot showing DS inhibits PI3K and mTORC1 and mTORC2 mediated activity.

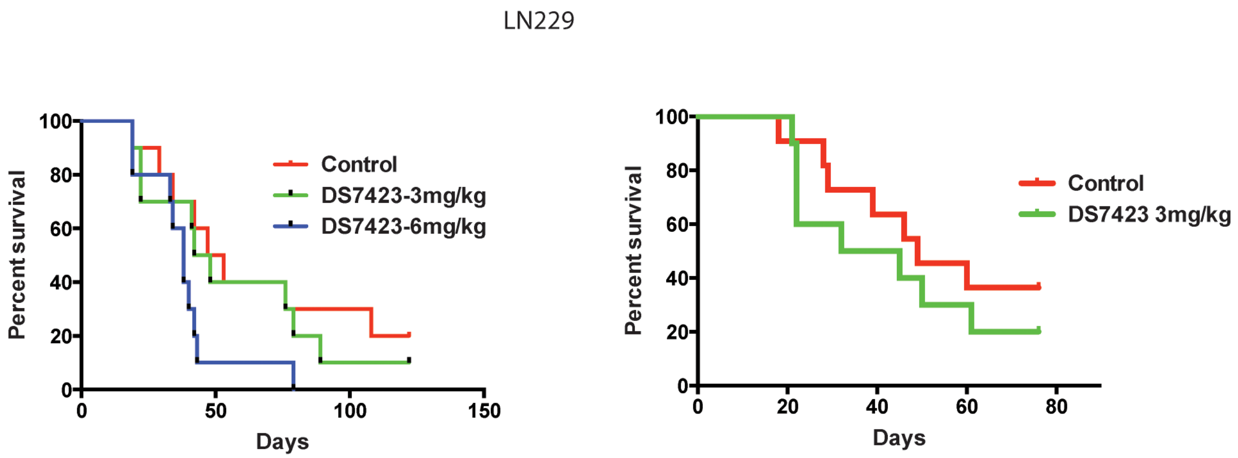

Supplemntary Figure 2: DS was ineffective in a LN229 tumor bearing mice and had no effect in median survival of animals clearly demonstrating that DS was ineffective in PTEN/PI3K wild type background. The study was repeated twice.
